# Quaternary history and contemporary patterns in a currently expanding species

**DOI:** 10.1186/1471-2148-9-220

**Published:** 2009-09-04

**Authors:** Carole Kerdelhué, Lorenzo Zane, Mauro Simonato, Paola Salvato, Jérôme Rousselet, Alain Roques, Andrea Battisti

**Affiliations:** 1 INRA, UMR1202 BIOGECO, F-33610 Cestas, France; 2 Dipartimento di Biologia, Università di Padova, 35121 Padova, Italy; 3 Dipartimento di Agronomia Ambientale e Produzioni Vegetali, Agripolis, Università di Padova, 35020 Legnaro PD, Italy; 4 INRA, UR633 Zoologie Forestière, F-45075 Orléans Cedex, France

## Abstract

**Background:**

Quaternary climatic oscillations had dramatic effects on species evolution. In northern latitudes, populations had to survive the coldest periods in refugial areas and recurrently colonized northern regions during interglacials. Such a history usually results in a loss of genetic diversity. Populations that did not experience glaciations, in contrast, probably maintained most of their ancestral genetic diversity. These characteristics dramatically affected the present-day distribution of genetic diversity and may influence the ability of species to cope with the current global changes. We conducted a range-wide study of mitochondrial genetic diversity in the pine processionary moth (*Thaumetopoea pityocampa*/*T. wilkinsoni *complex, Notodontidae), a forest pest occurring around the Mediterranean Basin and in southern Europe. This species is responding to the current climate change by rapid natural range expansion and can also be accidentally transported by humans. Our aim was to assess if Quaternary climatic oscillations had a different effect across the species' range and to determine if genetic footprints of contemporary processes can be identified in areas of recent introduction.

**Results:**

We identified three main clades that were spatially structured. In most of Europe, the genetic diversity pattern was typical for species that experienced marked glaciation cycles. Except in refugia, European populations were characterized by the occurrence of one main haplotype and by a strong reduction in genetic diversity, which is expected in regions that were rapidly re-colonized when climatic conditions improved. In contrast, all other sub-clades around the Mediterranean Basin occurred in limited parts of the range and were strongly structured in space, as is expected in regions in which the impact of glaciations was limited. In such places, genetic diversity was retained in most populations, and almost all haplotypes were endemic. This pattern was extreme on remote Mediterranean islands (Crete, Cyprus, Corsica) where highly differentiated, endemic haplotypes were found. Recent introductions were typified by the existence of closely-related haplotypes in geographically distant populations, which is difficult to detect in most of Europe because of a lack of overall genetic structure.

**Conclusion:**

In regions that were not prone to marked glaciations, recent moth introductions/expansions could be detected due to the existence of a strong spatial genetic structure. In contrast, in regions that experienced the most intense Quaternary climatic oscillations, the natural populations are not genetically structured, and contemporary patterns of population expansion remain undetected.

## Background

Past climate changes have had dramatic impact on the geographic distribution, demography, and thus the evolution of species. The contemporary distribution of genetic diversity cannot be understood without studying how organisms responded to climate over geological times. Many terrestrial species are today responding to the contemporary global warming [1], and their future response will at least partially depend on their previous reactions to climatic oscillations. The 'genetic legacy of the Quaternary ice ages' [2], *i.e*. the genetic footprint of species' responses to glacial-interglacial successions, has been extensively studied on many species in Europe and North-America, that is, in the geographical regions where glaciations were most intense [3,4]. Forest insect herbivores, such as those associated with oaks and pines in Europe and the Mediterranean, for example, are known to have responded to post-glacial warming with rapid range expansion northwards and eventually westwards, and to have survived glaciations in southern refugia [5-10]. The intensity of the oscillations increased with latitude, which affected the impact they had on species occurring through a gradient in the so-called ORD (Orbitally forced species Range Dynamics: see [11]).

Following Pinho and collaborators [12], we can make two predictions. In northern latitudes, where the effects of glaciations were more severe, fewer and smaller patches of suitable habitat were left for the survival of populations across multiple glaciation cycles, which would have resulted in overall lower diversity, and a lower number of differentiated lineages in northern than in southern areas. Moreover, the effects of climatic changes on the effective population sizes were more dramatic in northern than in southern regions, meaning that northern populations should bear the signature of a rapid demographic expansion following the climate amelioration, whereas southern populations should evidence marks of more stable, long-term effective population sizes.

Going further, Dynesius and Jansson [11] have predicted differential evolutionary consequences depending on the intensity of the ORD, and these predictions were empirically demonstrated for some taxa. Species that survived a strong ORD during the Quaternary, *i.e. *species occurring at higher latitudes, were selected for increased vagility and generalism. Dispersal-related traits should have been optimized during the northward progression because high mobility provided an elevated fitness within populations that were tracking a moving habitat. In the same way, generalists (in terms of habitat, host, or diet) had a smaller risk of their niche disappearing. Over evolutionary times, the selective pressures are likely to have changed, with dispersion and generalism favoured during interglacials, and less so during glacial periods when the species were restricted to suitable refugia.

The effects of differential intensities of glaciations on the evolution of the species, described above, are expected for mainland species for which the tracking of acceptable environments through migration was possible. The situation was drastically different for species or populations on islands situated beyond dispersal range, for which any change had to be endured locally, either by altitudinal shifts or by the evolution of local adaptations. Moreover, smaller effective population sizes could have resulted in loss of genetic diversity due to genetic drift. In this case, evolution on islands may have been more rapid than the rate of change on continents [13], and island populations are thus expected to be highly differentiated from both a genetic and an ecological point of view.

Species or populations that experienced marked climatic oscillations in the past can be seen as a selected assemblage of geographically mobile and latitudinally-independent organisms that are likely to be best adapted for the future climate changes, unless human activity precludes such an option [13]. Yet, comparing the phylogeographic patterns of species occurring over a latitudinal gradient is not straightforward, as other important factors such as life-history traits, ecological requirements, and dispersal ability will probably differ among species. Moreover, data on current modifications to distribution ranges due to global changes are also required to link differential Quaternary histories to present-day evolution.

**Figure 1:**
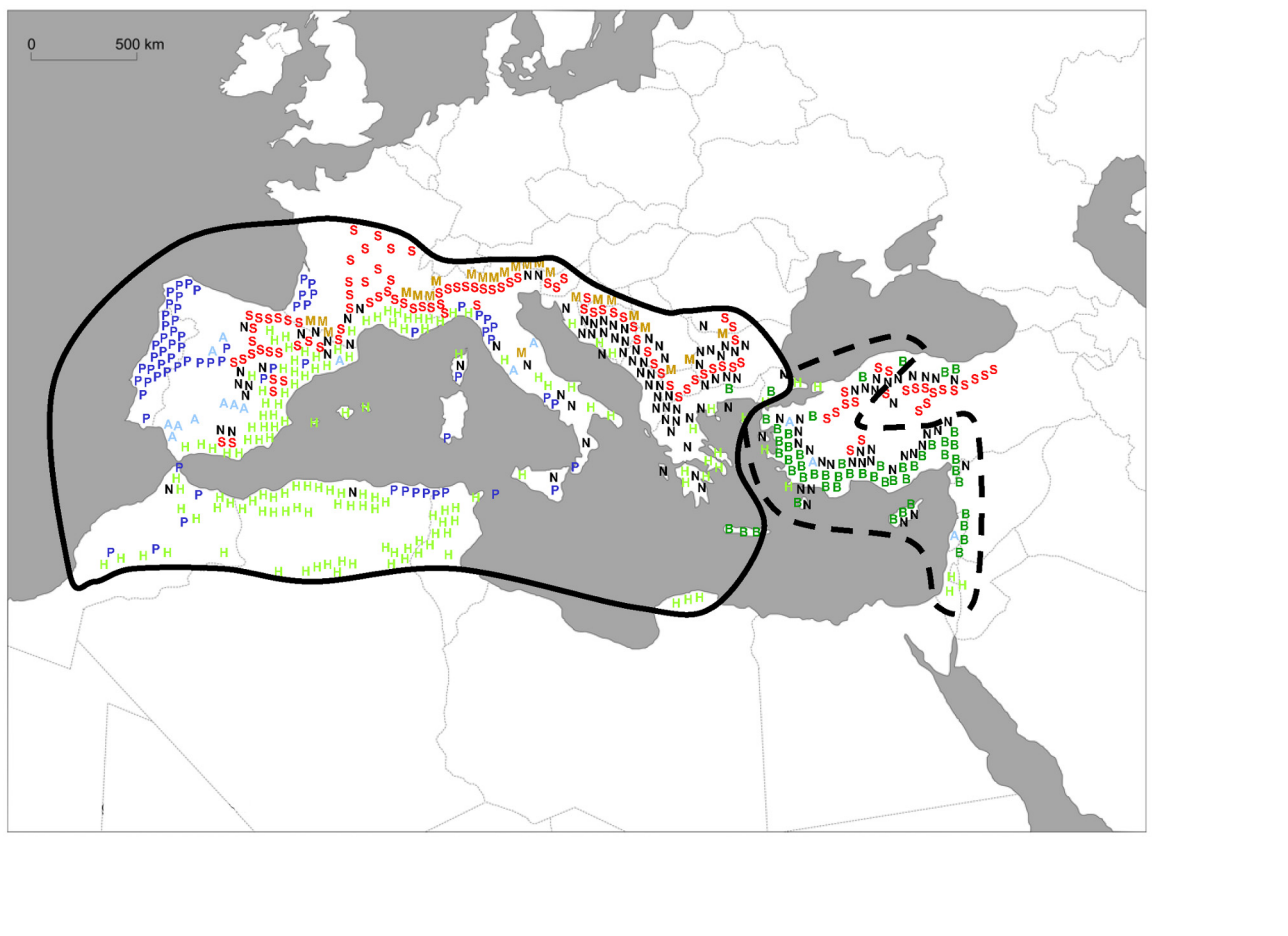
**Ranges of the pine processionary moths indicating the occurrence of native *Pinus***. *Thaumetopoea pityocampa*, solid line; *Thaumetopoea wilkinsoni*, dashed line; A = *Pinus pinea*, B = *P. brutia*, H = *P. halepensis*, M = *P. mugo*, N = *P. nigra*, P = *P. pinaster*, S = *P. sylvestris*. Each letter refers to a land unit where the indicated pine species is dominant but not necessarily exclusive. Other pine species may occur in the same area. *Thaumetopoea *distribution was drawn from: Anonymous (1977) Pest: *Thaumetopoea pityocampa *(Schiff.) (Lep., Notodontidae) (Pine processionary moth). *Distribution Maps of Pests, CAB*, **366**, 1-2. and *Pinus *distribution from: Richardson DM (1998) *Ecology and Biogeography of Pinus*. Cambridge University Press, Cambridge, UK.

Here, we present a range-wide genetic study of a circum-Mediterranean insect taxon: the winter pine processionary moth (*Thaumetopoea pityocampa*/*wilkinsoni *species complex), which develops mainly on pine species (*Pinus *spp.). It is a serious forest pest as it can cause heavy defoliations of pines in Mediterranean countries. *T. pityocampa *has a typical winter larval development [14]. Adults lay eggs on pine leaves in summer, and larvae feed from needles during fall and winter. They pupate in the soil in late winter or early spring, and newly emerged adults disperse to reproduce during summer. Larvae are gregarious and develop in a typical silk shelter. Ecological and genetic data based on mitochondrial and nuclear markers suggest that the species exhibits clear sex-biased dispersal, as females are poorer fliers than males [15,16]. It is present on both the northern and southern rims of the Mediterranean Basin as well as in the Middle-East (Figure 1), that is, in regions where the impacts of glaciations varied in intensity. Glacial cycles were probably most intense in temperate Europe, while ice sheets are believed not to have occurred in southern Mediterranean countries, nor in the Near East. Populations of the pine processionary moth are currently believed to belong to a species complex including two congeneric taxa: *Thaumetopoea pityocampa *and *T. wilkinsoni*. The differentiation between these two species was recently shown [17], and the monophyly of *T. wilkinsoni *populations in the near East was confirmed [16].

Current global changes can affect the genetic patterns of the pine processionary moth in different ways, and superimpose new signatures on existing natural phylogeographical structure. An increase in mean winter temperatures in Europe is known to drive moth expansion northward and to higher altitudes, in regions where hosts are available, by providing suitable conditions in places were larvae could not previously have survived [18]. And if environmental conditions are suitable for the insect's development, new pine plantations can also increase the potential range of the pest by offering hosts in places where they were not previously available. Contemporary changes in the moth's distribution range can proceed either from a natural, non-assisted expansion of insect populations into newly suitable habitats, or from long-range dispersal that is likely to be human-aided (accidental transportation of adults or larvae, or transplantation of buried pupae when mature trees are planted). In cases of natural expansion, we expect a gradual loss of diversity away from the native range (e.g., [10,19]), while long-distance, assisted introductions should result in a discontinuous distribution of genetic diversity. A recent study of the range-wide genetic structure of the oak gall-wasp *Andricus kollari *showed that the patterns observed in England were consistent with the hypothesis of man-aided, long-distance introductions [7].

The aim of our study was to infer the Quaternary history of the species complex over its whole distribution range, to test if the effects of Quaternary climatic oscillations can be differentially detected in the different parts of the range, and if any impact of global change can be detected and interpreted in the light of the species' evolutionary history. Both mitochondrial and nuclear markers are useful to reconstruct the evolutionary history of a species complex. Although nuclear markers such as AFLPs and microsatellites were previously developed for this species [15-17], we were not able to use them in this range-wide study because of homoplasy and because of the occurrence of null alleles in divergent clades. We thus present data based on mitochondrial DNA alone. As female dispersion is the limiting factor for species expansion, inferring the history of female lineages provides a good indication of species dispersal. Yet, potential biases due to the use of mitochondrial markers alone, such as the selective sweep that can be caused by bacterial symbionts [20], as well as the limits inherent to single gene phylogenies, should be acknowledged.

## Results

We obtained 34 COI and 51 COII haplotypes. Among these, 14 COI and 21 COII haplotypes were known from either Salvato et al. [17] or Simonato et al. [16] and were already available in GenBank (accession numbers EF015538-EF015549 and EF210075-EF210097). The new haplotypes found in the present study have been deposited in GenBank (accession numbers GQ507373 to GQ507422). A total of 67 combined (COI-COII) haplotypes (ht) were found. The selected model of evolution was the General Time Reversible model with gamma distributed heterogeneity of rates (GTR gamma). Interestingly, Bayes factors (BF) indicated a much stronger fit for this model when a clock was assumed than when branch lengths were unconstrained (BF = 142, computed as twice the difference in logarithm of harmonic means of likelihoods). This was confirmed when the performance of models was assessed with the Bollback approach [21]. The GTR gamma model was then used for all subsequent analyses. The specific rates were A-C: 0.144; A-G: 1.166; A-T: 0.068; C-G: 0.031; G-T: 0.019 and α = 0.152.

### Phylogenetic inference and node datation

**Figure 2:**
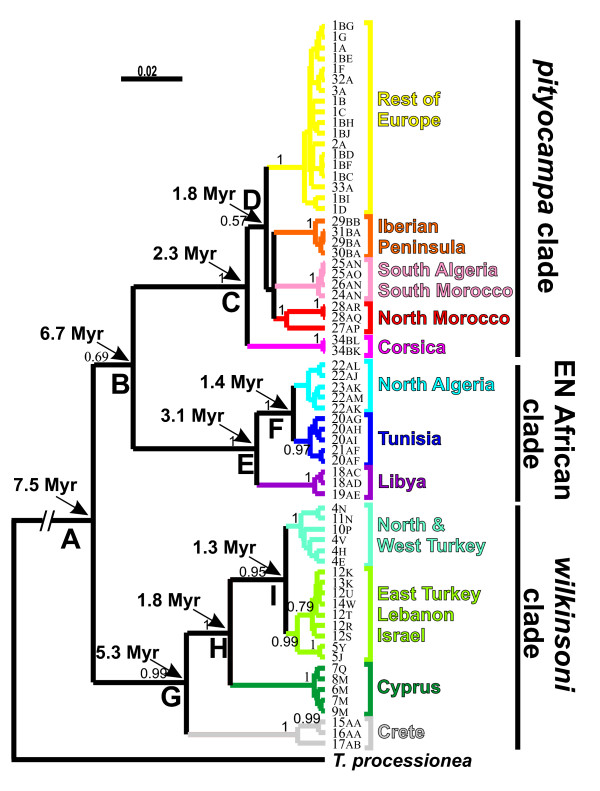
**Bayesian consensus tree for all Mediterranean *Thaumetopoea pityocampa *and *T. wilkinsoni *haplotypes rooted on *T. processionea***. Bayesian supports over 0.5 are given. The arrows show the estimated age of the most recent common ancestors (in million years) of the deeper supported nodes. Age estimates and their corresponding 95% confidence intervals are given in Tables 1 & 2.

The haplotype composition of each sampled population is given in Additional file 1 (Sampling sites, geographic coordinates, host pine, collector and haplotype composition of each locality). The phylogenetic analysis clearly showed that the *T. pityocampa - wilkinsoni *complex was structured in three strongly supported clades (Figure 2). A first group of 23 ht clustered all sequences corresponding to *T. wilkinsoni *[16] together with the ht found on the island of Crete. This '*wilkinsoni *clade' was the sister group of all other ht. A second clade of 13 ht was restricted to Libya, Tunisia (including the nearby Italian island of Pantelleria) and North Algeria ('Eastern North Africa clade', hereafter ENA clade). The third clade comprised 24 European ht, from Spain and Portugal to Greece (with the notable exception of Crete), together with the 7 ht found in Morocco and South Algeria. It will hereafter be referred to as the '*pityocampa *clade'. The main nodes were dated by Bayesian inference using a Yule prior and the estimates are given on the phylogenetic tree (Figure 2) and in Table 1, with 95% confidence intervals (CI). The split between the *wilkinsoni *clade and the 2 others was ca. 7.5 Million years ago (Myrs; 95% CI 5.8 - 9.3), while the separation of the *pityocampa *vs. ENA clades was dated back to 6.7 Myrs (4.9 - 8.6). The age of the most recent common ancestor (MRCA) of the *wilkinsoni *clade was estimated to 5.3 Myrs (3.7 - 7.1) while that of the ENA clade was ca. 3.1 Myrs (2.1 - 4.3) and that of the *pityocampa *clade was estimated to 2.3 Myrs (1.6 - 3.1).

**Table 1:** Age estimates of phylogenetic tree nodes and 95% confidence intervals.

**Node code**	**Estimated age of the node (in Myrs)**	**95% confidence interval (in Myrs)**
**A**	7.450	5.776 - 9.271
**B**	6.742	4.892 - 8.613
**C**	2.348	1.631 - 3.124
**D**	1.772	0.921 - 2.725
**E**	3.146	2.104 - 4.298
**F**	1.364	0.766- 2.025
**G**	5.332	3.688 - 7.067
**H**	1.846	1.210 - 2.545
**I**	1.259	0.742 - 1.060

Estimations were performed by analysing all the haplotypes and assuming a Yule prior. The node codes are given in Figure 2.

**Figure 3:**
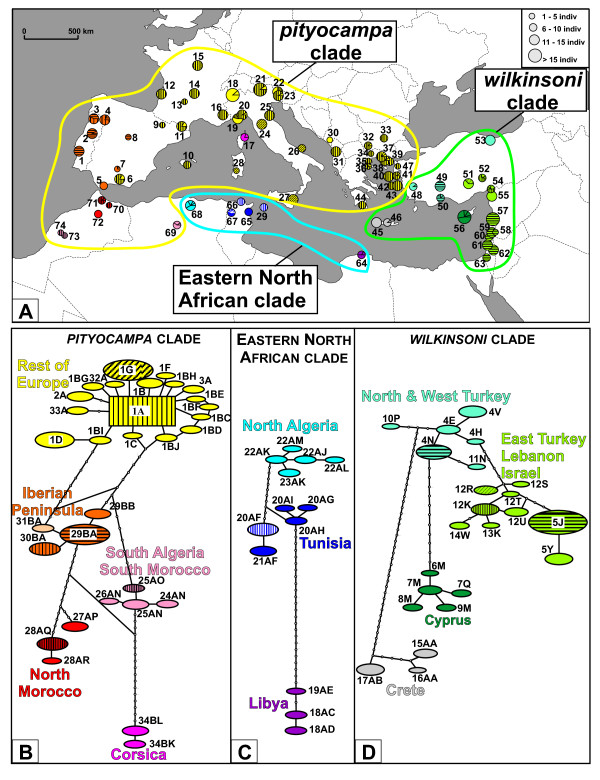
**Geographical distribution of mitochondrial haplotypes of the *Thaumetopoea pityocampa/T. wilkinsoni *complex, and within-clade haplotype networks**. A. Geographical mapping of haplotypes in the sampled populations. Circles are proportional to the number of individuals analyzed in each population and colors refer to the major clades identified in network analyses. Codes of populations are given in Additional file 1. B. Haplotype network of the '*pityocampa' *clade. Each line in the network represents a single mutational change. Empty circles indicate intermediate, missing haplotypes. C. Haplotype network of the 'Eastern North Africa' clade. D. Haplotype network of the '*wilkinsoni' *clade.

Further geographic structure was found within the three main clades. In the *wilkinsoni *clade, 4 distinct sub-clades were found with very high support values (Figure 2). The Cretan sub-clade formed the sister group of all other ht. The Cypriot ht were the sister group of the North & West Turkey sub-clade and of the sub-clade grouping the ht from East Turkey, Lebanon and Israel. The ENA clade was divided in 3 sub-clades corresponding to the 3 countries in which the larvae were sampled. The Libyan ht formed a sister group relative to the North Algerian and to the Tunisian sub-clades. Finally, the *pityocampa *clade was comprised of five strongly supported geographical groups. Haplotypes from the island of Corsica appeared as the sister-group of the four remaining sub-clades: (i) the Iberian Peninsula, (ii) North Morocco, (iii) South Morocco & South Algeria and (iv) the Rest of Europe. Interestingly, 16 individuals sampled in Spain and 2 individuals from western Turkey had "Rest of Europe" haplotypes (Figure 3).

Time of most recent ancestor (tMRCA) of each sub-clade was estimated by Bayesian inference including all the individuals of a given group and assuming a priori either a constant population size or an exponential growth. However, the rate of exponential growth resulted to be positive, with an associated 95% confidence interval excluding 0, for the Rest of Europe only (g = 6.2 10^-5 ^yrs^-1^; 95% CI: 6.2 10^-6 ^- 1.4 10^-1 ^yrs^-1^); tMRCAs obtained with an exponential prior are reported only for this group (Table 2). Keeping in mind that our use of a rate from phylogenetics studies will bias estimates upwards, estimated ages for tMRCA resulted to range from 532 000 years ago for North Morocco to 90 000 years ago for Rest of Europe.

**Table 2:** Estimates of tMRCAs of the most recent nodes (main sub-clades) and 95% confidence intervals.

**Sub-clade**	**tMRCA (in Myrs)**	**95% confidence interval (in Myrs)**	**Tree Prior**
**Rest of Europe**	0.090	0.028 - 0.172	Exponential*
**Iberian Peninsula**	0.091	0.005 - 0.201	Constant
**South Algeria - South Morocco**	0.130	0.011 - 0.290	Constant
**North Morocco**	0.532	0.194 - 0.905	Constant
**Corsica**	--**		Constant
**North Algeria**	0.171	0.026 - 0.355	Constant
**Tunisia**	0.326	0.092 - 0.601	Constant
**Libya**	--**		Constant
**N & W Turkey**	0.332	0.114 - 0.608	Constant
**E. Turkey, Lebanon, Israel**	0.417	0.148 - 0.711	Constant
**Cyprus**	0.151	0.021 - 0.313	Constant
**Crete**	0.381	0.116 - 0.688	Constant

Estimates were obtained by assuming a coalescent prior of constant size or exponential growth and by including all the sequences of each given sub-clade. Names are the same as in Figure 2.

* Results based on an exponential prior are reported because the rate of exponential growth (g) was significantly higher than 0 for this group.

** MCMC did not converge due to small sample size (N = 10 and 6 for Corsica and Libya, respectively)

### Haplotype distribution and haplotype network

Haplotype networks were reconstructed for each of the 3 main clades, and haplotype distributions were mapped (Figure 3). The haplotype networks recovered the same strong geographical patterns as the phylogenetic tree. Within the *wilkinsoni *clade, most ht were found in a single population, except ht 5J (found throughout Lebanon and Israel and shared by 91 individuals), and ht 4N, 12R and 12K that each occurred in two populations (see Additional file 1, sampling sites, geographic coordinates, host pine, collector and haplotype composition of each locality). All but one ht (20AF, found in Tunisia and Pantelleria) in the ENA clade were endemic to one population. Finally, the *pityocampa *clade was divided into the 5 sub-clades found on the phylogenetic tree. The network of the Rest of Europe sub-clade was star-shaped (which is typical for expanding populations), with one main ht shared by ca. 74% of sampled individuals and all other ht diverging from it by only one or two mutations. Haplotype 1G was restricted to central and southern Italy. Interestingly, all other haplotypes in Europe were rare, shared by 6 individuals at most and usually endemic to one population. In the Iberian Peninsula, ht 29BA was found in 57% of individuals and in all populations but Gibraltar and two southern sites. None of the other sub-clades in the *pityocampa *group showed a star shape.

Within population, gene diversity H and nucleotide diversity per site π are given in Additional file 1. Fu's Fs and Tajima's D were estimated and tested within each of the 12 sub-clades except for the Libyan group as it was composed of only 6 individuals. Both indices were significantly negative only in the Rest of Europe sub-clade (see Figures 2 &3 and Table 3). Mismatch analyses were consistent with the sudden expansion model for this sub-clade (SSD = 0.00298, P = 0.746) and showed a unimodal distribution that closely fit the expected distribution. In this sub-clade, τ was estimated to 1.77 (95% CI: 0 - 4.20), and the corresponding expansion was thus estimated to date back ca. 147 000 years (95% CI: 0 - 348 261 years).

**Table 3:** Indices of genetic diversity per identified sub-clade, Tajima's D and Fu's Fs statistics

**Sub-clade**	**N**	**Hd**	**π**	**Tajima's D**	**Fu's Fs**
Corsica	10	0.36	0.06%	0.01 NS	0.42 NS
South Algeria - South Morocco	13	0.73	0.15%	- 0.14 NS	- 0.69 NS
North Morocco	24	0.55	0.69%	2.35 NS	7.22 NS
Iberian Peninsula	61	0.60	0.12%	0.18 NS	0.10 NS
Rest of Europe	358	0.44	0.11%	- 1.82 **	- 15.82 **
North Algeria	12	0.79	0.19%	- 0.54 NS	- 1.61 NS
Tunisia	30	0.62	0.30%	0.10 NS	1.04 NS
Libya	6	-	-	-	-
Crete	21	0.55	0.52%	2.03 NS	5.26 NS
Cyprus	19	0.72	0.15%	-0.60 NS	- 1.42 NS
North & West Turkey	45	0.68	0.20%	- 0.93 NS	- 0.56 NS
East Turkey, Lebanon, Israel	133	0.51	0.35%	- 0.10 NS	0.44 NS

N: # individuals; Hd: gene diversity; π: nucleotide diversity per site. NS: non significant; **: p < 0.001. The names of the sub-clades are the same as in Figure 2.

## Discussion

### Overall phylogenetic patterns around the Mediterranean Basin

The pine processionary moth is currently understood to consist of a species complex containing two taxa, namely *Thaumetopoea pityocampa *and *T. wilkinsoni *[16,17]. Surprisingly, the thorough sampling we obtained clearly proved that the species complex is composed of three rather than two main clades, as the populations from ENA appeared as the monophyletic sister group of the *pityocampa *clade (Figure 2), and the *wilkinsoni *clade (including populations from Crete) as the sister group of the ENA and *pityocampa *clades. Determining the taxonomic status of the clusters identified here is beyond the scope of the present study, and would need complementary data such as nuclear markers and morphological data. For this reason, we will hereafter mention three clades (the *pityocampa *clade, the ENA clade and the *wilkinsoni *clade) without further discussion of their taxonomic level.

Another striking result is that the species complex is ancient, and predates the Quaternary by a few million years. In a previous study, the divergence between *Thaumetopoea pityocampa *and *T. wilkinsoni *was estimated to 4.5 - 5.2 Myrs [17]. That result was obtained from a limited sampling, in which only the European clade of *T. pityocampa *(as the population from Spain contained only European haplotypes rather than Iberian ones) and one Turkish population of *T. wilkinsoni *were analyzed. In the study presented here, a thorough sampling of populations (including the Cretan lineage of *wilkinsoni*, as well as most sub-clades of *pityocampa *and the previously unknown ENA clade) and a Bayesian approach taking into account the gamma-distributed heterogeneity of rates, allowed us to obtain a different estimate for the age of the main evolutionary events. In particular, the split between the *wilkinsoni *and the *pityocampa*-ENA clades was dated on average to 7.5 Myrs, with a confidence interval of 5.8 - 9.3 Myrs, which could correspond to the full opening of the Aegean Trench ca. 9 Myrs ago [4,22]. Interestingly, within the *wilkinsoni *clade, the estimates of node ages we obtained were very similar to estimates obtained previously using codon-partitioned models [16]. While we did not have enough a priori evidence to calibrate our own molecular clock, it should be noted that, by using the universal rate, the divergence of Crete from all the other *wilkinsoni *haplotypes was dated back to about 5.3 Myrs, which corresponds to the Messinian salinity crisis and the time when the Mediterranean Sea was at its lowest level, thus making the colonization of islands easier [23]. Node ages should, however, always be interpreted with caution, given that a single mitochondrial locus was used [20].

The differentiation between the *pityocampa *and the ENA clades was unexpected, and cannot be explained by classical barriers to gene flow such as mountain ranges or fragmentation of suitable habitats. Similar patterns of East-West genetic differentiation have occasionally been found in North Africa for other organisms [24-27], but were estimated to date back to various times, from 1.6 to 12 Myrs. A range of hypotheses have been proposed by the authors to explain the abrupt genetic differentiation within species in this region. They invoked either climatic scenarios, with the rapid alternations of arid and humid periods acting as a spatially structuring force in this region during the Quaternary; or biogeographical scenarios such as the formation of the Straits of Gibraltar after the Messinian salinity crisis, the split of the Tellian (Tell) Atlas at the Sicilian Channel, or the more ancient formation of the Neo-Pyrenees. Indeed, the pine processionary moth depends on the presence of pine hosts for development, and it is known to be susceptible to summer aridity and excessive heat [28]. Moreover, it was recently shown that barriers of moderate altitude can hamper gene flow in this species [29]. Finally, the species also exhibits large among-population variation in term of reproductive phenology [28] that permits the adaptation of populations to the local climatic conditions and may also limit gene flow. Thus, the conjunction of major biogeographical events (the rise of the Tellian Atlas) and late Tertiary climatic change (with a possible gap in host availability during more arid phases) could explain the split that occurred between the *pityocampa *and the ENA clades some 6-7 Myrs ago.

If the main divergences within the *T. pityocampa*/*wilkinsoni *complex date from the end of the Miocene, all clades also predate the Quaternary. Each of the identified clades thus experienced the Quaternary climatic oscillations after they split from a common ancestor, and the impact of ice ages can easily be compared between these closely-related clusters.

### Phylogeographical patterns and within-clade structures

Each of the three identified clades showed a strong phylogeographical structure, and was composed of 3, 4 or 5 well-differentiated sub-clades. With the notable exception of the Rest of Europe (see below), each sub-clade was restricted to a rather narrow geographical region. Interestingly, a vast majority of haplotypes (54 out of 67) were endemic to one single population, and only five were found in three or more populations. Thus, the pine processionary moth exhibits an extreme spatial structure and a highly reduced mitochondrial female gene flow even on a regional scale, even though results based solely on a mitochondrial marker should be interpreted with caution. Over most of the distribution range, the actual dispersal of the females is thus highly limited. The main barriers to gene flow are sea straits, mountain ranges (the Pyrenees, Taurus Mountains, High Atlas, Saharian Atlas), or desert regions where hosts are lacking (Libya).

Within-clade structures were all dated back to at least 1.3 Myrs (Figure 2), *i.e. *to the Early- or Mid- Pleistocene. One could suggest that local ecological pressures recurrently acted to reinforce and maintain the genetic structures whenever gene flow had been interrupted. As migration is very limited and cannot counteract the effects of drift, genetic differentiation then simply increases with time, leading to divergent lineages in different regions. Ecological factors involved in differentiation include reproductive phenology, which can prevent mating by shifting adult emergence periods in different populations, or local adaptation to host characteristics, which, it has been proved, can lead to complete mortality in translocated larvae [30]. A more precise sampling in North Africa would allow the delimitation of the exact distribution ranges of each sub-clade, and the determination of whether contact zones do exist between them.

Once again with the exception of the Rest of Europe sub-clade, a majority of the sampled populations in the natural area of the species show more than one haplotype, even when only 5-10 individuals were sampled, and even at the edge of the distribution or in very isolated places such as Libya or on remote islands. Like many insects, the processionary moth has evolved the capacity of prolonged diapause, which allows the emergence of adults of the same generation over several years, thus limiting the risk of local extinction and increasing the probability of retaining local genetic diversity. A high genetic diversity in the southernmost populations has also been observed for other Mediterranean insect species (e.g., [8,9,31,32]). Interestingly, no sign of demographic expansion could be detected in these regions, as is expected in regions where glaciations were less intense [12]. However, one region in the Near East is characterized by an extreme genetic depauperization as one single haplotype is present in Lebanon and Israel. This is probably linked to the very recent origin of moth populations in this region, where pine trees were not present before the beginning of the XX^th ^century except for remote relictual stands (see Simonato et al. [16] for a detailed discussion). The moth has expanded slowly following afforestation. Recent expansions due to global changes are discussed below.

Europe (except the Iberian Peninsula that harbours a specific sub-clade) is characterized by a major haplotype that occurs from the Atlantic coast to the Greek islands and even along the Turkish border. Moreover, the Rest of Europe sub-clade had the star-shape that is typical for populations expanding after a demographic bottleneck [33], and the Bayesian analyses indicated for this group a positive exponential growth supporting a past demographic expansion [34,35]. Tajima's D and Fu's Fs statistics revealed an excess of rare haplotypes and allowed us to reject mutation-drift equilibrium. As similar results can be obtained from different processes (see for instance [36,37]), we conducted a mismatch analysis that also indicated that European populations underwent bottleneck events due to the recurrent glaciation periods and then recurrently expanded after the retreat of the ice. Such results are classically found for temperate and cold-sensitive species in this region [4,9,10]. The spatial distribution of the rare haplotypes gives insights into the existence and locality of refugial areas where the moths survived the glaciations, and possibly also the interglacials as this Mediterranean species is susceptible to both winter cold and summer heat and aridity [28]. As for most of the European temperate species, these moth refugia are located in the Balkans and in Italy, as well as in the western part of the Iberian Peninsula [4]. Our results also show that the Alps and the North of Italy form a region with a high proportion of endemic haplotypes, thus differing from all other regions in Europe. This could indicate that this area also was a Quaternary refugium where part of the ancestral polymorphism was locally retained. Interestingly, the Alpine Arc was recently proved to be a refugial area for *Pinus sylvestris *[38], which suggests that the refugial moth populations could have survived the glacial maxima in this region on that particular host.

With the exception of Lebanon and Israel where the moth settled and expanded only recently (see below), our results show contrasting patterns of evolution during the Quaternary in the different regions of the moth's distribution range, corresponding to our expectations. In particular, populations occurring in the highest latitudes exhibit a radically different genetic footprint to that of all other sub-clades. If moth populations in the vast majority of the distribution range are characterized by a strong spatial genetic structure, a high number of endemic haplotypes and a restricted geographical range for each identified sub-clade, the patterns in the Rest of Europe are completely the opposite. In this European region, overall genetic diversity is low; spatial genetic structure is limited as a consequence of the large distribution of the major haplotype 1A; and this single sub-clade is distributed over one half of the total distribution area of the species complex. Moreover, signs of recent expansion were detected only in the European sub-clade, that is, in the region where glacial cycles were probably most intense. As for most European species, endemic haplotypes and some genetic variability can still be detected in plausible refugial areas near the Pyrenees, in Italy and in the Balkans [4,8,13]. In the rest of the area, the recurrent northward expansions that followed climate warming after glacial maxima were probably rapid, pioneer-like [39], and lead to a genetic homogenization of populations. In other temperate forest insect species, genetic diversity was also mostly retained either in the southernmost populations [9,31], or in the eastern regions where the impact of the Quaternary cycles was less pronounced (as for *Andricus *gall wasps developing on oaks, see [5,6,32]).

### Evolution of insular populations

In each of the three main clades, the most divergent sub-clade corresponds to an island, or to an island-like continental region. The Corsican ht are the most differentiated within the *pityocampa *clade, the Cretan ht form the sister-group of all other sub-clades within the *wilkinsoni *clade, and the highly isolated moths of Cyrenaica (Libya) are most divergent in the ENA clade. Moreover, the second most differentiated group in the *wilkinsoni *clade is the Cypriot cluster. Each of the island lineages thus diverged from the corresponding sub-clade a long time ago (from 5.3 Myrs for the Cretan haplotypes to 1.8 Myrs for Cyprus). On the other hand, the most recent common ancestors for each island are much more recent (0.38 Myrs in Crete and 0.15 Myrs in Cyprus for example). Hence, it is not possible at this point to determine when exactly the colonization of each island (or isolated place) occurred, and for how long the moths have been isolated from the continent. However, even if we consider only the estimated age of the MRCA (which could be overestimated because we used a rate from phylogenetic studies, see [40], though the use of a Bayesian coalescent prior should in part address this problem), we can suggest that the pine processionary moths survived locally on these remote islands without female exchanges from the continent during few glacial cycles. As a consequence, they had to evolve locally to cope with at least some Quaternary oscillations and environmental changes [13]. The quite recent estimate for the age of MRCA for each island could be due to a founder effect followed by the effect of genetic drift in small populations [5], as well as by fixation of selected variation. We have evidence, in the pine processionary moth, that male gene flow have occurred between Cyprus and the continent [16], as was suggested by the strong genetic similarity between Cypriot and Turkish populations found with both AFLPs and microsatellite markers. This could also be true for islands situated at moderate distance from the continent.

### Contemporary patterns in a historical context

In recent years, the distribution range of the processionary moth has been affected by global changes, mainly through winter warming [18] and pine afforestation. Moreover, it is suspected that human-aided dispersal occurs over various distances, either via 'hitch-hiking' (passive transportation of individuals) or accidental transplantation of pupae with grown trees moved with a substantial amount of soil. The genetic signatures of these contemporary events will be different, and may not be easy to detect in all regions. In most regions around the Mediterranean Basin, apart from Europe, the natural phylogeographic pattern consists of genetically diverse and spatially structured populations. Regions with surprisingly low levels of genetic diversity (e.g. Lebanon and Israel), or sampling sites that are genetically closely related to geographically distant populations (e.g. site 53 in Turkey, or 69 in Algeria) can be easily identified. These sites actually correspond to zones of recent moth expansion either following anthropogenic pine expansion, such as in Israel or Algeria where pines were planted both in the beginning and at the end of the XX^th ^century, or following the ongoing climate warming that allows insects to survive winter in places where they could not some decades ago (site 53 near the Black Sea). Given the natural spatial genetic structure in these regions, the recent modifications in moth distributions due to global changes are actually easy to track. The populations discussed above all likely originated from the closest natural stand, and could be the result of non-assisted moth expansion (but a better sampling in Algeria is needed to confirm this). The mitochondrial marker we used here would also be useful to identify between-subclades female gene flow, but a nuclear marker is necessary to track male exchanges. In most of Europe, however, where the populations are not genetically structured in space and where overall genetic diversity is low, probably as a consequence of Quaternary history, one cannot distinguish recent and historical events, as contemporary expansions (proved at both higher latitudes and altitudes, see [18]) result in the loss of genetic diversity, as in the case of rapid, leptokurtic dispersal northwards that allowed re-colonization of northern habitats during interglacials [10,19].

The patterns are somewhat different for islands. Some harbour populations of moths that are genetically very close, or even similar, to their closest continental neighbours. This is not surprising for islands that are located very close to the continent, like most Greek islands or Sicily, that can probably be recurrently colonized from mainland sources. A similar result was found, for example, for rodents [41]. In contrast, one would expect the populations of Sardinia, Pantelleria, or the Balearic Islands, that are beyond the natural dispersal range, to be highly differentiated, as are the moths from Corsica, Cyprus or Crete. In Sardinia, pines are still very rare and, until recently, no pine processionary moths were found on the island. In 2004-2005, pines were transplanted from Tuscany and a population of the moth was detected the following year [42]. Not surprisingly, the moths sampled in Sardinia all bore the haplotype found in Tuscany, showing that the pests were accidentally introduced with their hosts. A similar hypothesis could be invoked to explain the occurrence of moths bearing the major haplotype 1A in the Balearic Islands, where the moth was first detected in the 1950s (G. Sanchez, pers. com.). The situation on the island of Pantelleria is different as genetic data show that pine trees (*Pinus pinaster*) occur naturally and exhibit a high degree of local genetic diversity [43]. In contrast to its pine host, the local moth population has low genetic diversity and bears the main Tunisian haplotype, suggesting that it was recently introduced.

## Conclusion

We conducted a range-wide study of genetic diversity in a species complex occurring across regions in which Quaternary oscillations differed in intensity - or were absent. We have clearly shown that the sub-clade distributed over Europe had a phylogeographical pattern typical for species that experienced marked glaciation cycles. Refugial areas, where genetic diversity was retained and where endemic haplotypes were found, were identified in Italy, in the Alps and in the Balkans. All other populations were characterized by the occurrence of one main haplotype and by a strong reduction in genetic diversity, as is expected in regions that were rapidly re-colonized by a limited number of migrants when climatic conditions improved. We have ecological evidence that the moth populations are currently experiencing an expansion due to global change (both climate warming and host plantations). However, in the temperate regions of Europe, the natural populations are not genetically structured in space. The contemporary patterns are thus indistinguishable from historical ones as they also consist in progressions of the most widely distributed haplotypes. In contrast, all other sub-clades occur in limited ranges and are strongly structured in space, as is expected in regions that did not experience Quaternary cycles of glaciations. In these areas, genetic diversity has been retained in most populations, and each haplotype is usually found in only one population. The genetic signatures of recent moth introductions/expansions in these regions can be easily detected: recent expansions are characterized by the loss of genetic diversity across whole regions (e.g. Lebanon and Israel), and recent introductions are typified by the existence of closely related haplotypes in geographically distant populations. A strong differentiation is also expected for island populations if the island colonization occurred naturally in geological times. Thus, the occurrence (or not) of a significant 'natural' genetic structure of populations will determine whether or not recent expansions or introductions can be detected in the genetic data.

Complementary data based on polymorphic nuclear sequences would now be useful to compare biparental and maternally inherited markers, and to detect how male dispersal may have influenced the global evolutionary history of the species. Finally, our findings could be interesting for pest control as individuals present in different clades or sub-clades may have evolved different ecological characteristics (dispersal ability, host adaptation, egg size, resistance to parasitoids or pathogens), which can affect pest management strategies. Phenotypic traits should now be measured within each phylogenetic clade and sub-clade and compared between regions to test this hypothesis.

## Methods

### Moth sampling

Eggs and larvae of *Thaumetopoea pityocampa *and *T. wilkinsoni *were collected in 51 different locations from 16 countries in Europe and around the Mediterranean Basin. In addition, data from the 9 populations studied in Salvato et al. [17] and the 14 populations from Simonato et al. [16] were updated with newly sampled individuals and used here. The complete data set thus consisted of 74 populations (see Additional file 1 and Figure 3). Two to 26 individuals were sampled per population following a protocol described elsewhere [16], except in one locality in Morocco where only one individual could be found.

### DNA protocols

DNA was extracted using a salting-out procedure [44]. Two mitochondrial DNA (mtDNA) fragments, corresponding to parts of the COI and COII genes, were amplified from 732 individuals and analyzed by SSCP, as described in Salvato et al. [17]. For each mobility class, 1-5 individuals were sequenced to check for the accuracy of SSCP analysis and to determine the corresponding haplotype. Sequences were aligned using ClustalX [45]. Sequences of COI (263 bp) and COII (341 bp) fragments were then concatenated, resulting in a 604 bp-long final alignment.

### Data analyses

A partition homogeneity test was performed for the COI and COII fragments using Paup*4b10 [46]. The test confirmed that these regions contained homogeneous signals (p = 0.15), allowing data to be pooled for further analyses.

Model selection was performed using a Bayesian framework, through comparison of Bayes factors [47]. In addition, model performance was assessed using a posterior predictive test [48]. Models tested were selected using a modified version of Hierarchy 1 in MrModeltest 2.2 [49], enforcing or not a molecular clock. Given the limited length of the fragment analyzed and the correlation between proportion of invariant sites and the parameter alpha of the gamma distribution [47], we decided not to consider the invariant+gamma models.

For Bayes factors calculation, likelihoods for a given model were estimated using MrBayes v3.1.2 [50], and harmonic means were used as estimators of the overall marginal likelihood of the model. Each MrBayes analysis was the result of two independent chains of 2.10^6 ^generations, incrementally heated with T = 0.15. Convergence was assessed by computing the potential scale reduction factor with *sump *in MrBayes. Differences between Bayes factors obtained from the different models tested, calculated as twice the difference in the logarithm of harmonic means of likelihoods, were compared with reference values from Kass and Raftery [51].

For model performance assessment we chose as discrepancy variable the multinomial test statistics [52]. Posterior predictive distribution was evaluated through Monte-Carlo simulations of 1,000 datasets for each model using posterior densities of model parameters (tree topology, branch lengths and substitution parameters) inferred by MrBayes. MAPPS software [21] was used for simulations. The discrepancy between observed test statistics and simulated predictive distributions in the various models was quantified using Bayesian p-values [48] and the L-criterion proposed by Laud and Ibrahim [53], both computed with MAPPS.

Relationships between haplotypes and molecular dating were estimated by Bayesian inference of phylogeny using Beast v1.4.8 [54]. The model of sequence evolution and clock assumptions followed the results obtained from previous analyses and a Yule prior on the tree was assumed [55,56]; Markov chain Monte Carlo (MCMC) was run for 10 million generations, results being logged every 1,000 generations. After discarding the first 10% of the chain, convergence was checked by monitoring traces of sampled parameters and effective sample size following authors' suggestions. Analyses were cross-checked with MrBayes and the time of the most recent common ancestor (tMRCA) of selected clades was determined, assuming a sequence divergence rate of 2% per million years [57], and reported as a mean value with 95% highest posterior density interval (HPD).

For the most recent nodes, demographic Bayesian analyses were performed separately for each of the identified sub-clades using Beast and including all the sequences of a given group. Assumptions and settings were the same as above, except that coalescent priors of constant size and of exponential growth were used instead of Yule priors, and that two MCMC runs of 100 million steps were performed. tMRCAs of recent sub-clades were estimated assuming a 2% divergence, and must therefore be interpreted as the maximum age for a given sub-clade [40].

The phylogenetic reconstructions allowed us to identify three highly supported monophyletic clades within which a statistical parsimony network was computed using TCS v1.21 [58]. Such a network estimates genes genealogies from DNA sequences following the method described in Templeton et al. [59].

Gene diversity H and nucleotide diversity per site π were calculated within populations and within previously identified sub-clades. To infer whether each sub-clade has experienced recent population expansions, Tajima's D and Fu's Fs statistics were calculated and tested with DnaSP 4.10 [60]. Mismatch distributions of the pairwise genetic differences [61] were then performed using Arlequin 3.1 [62] and their goodness-of-fit to a sudden expansion model was tested using parametric bootstrap approaches (1000 replicates). The sum of squared deviations (SSD) between the observed and expected mismatch distributions was used to assess the significance of the test. Mismatch analyses were also used to estimate the approximate timing of expansion in the sub-clades where mutation-drift equilibrium was rejected. We used the relationship τ = 2*ut *[61], τ being the age of expansion measured in units of mutational time, *t *the expansion time in number of generations, and *u *the mutation rate per sequence and per generation. This last value was calculated using the relationship *u *= 2 μ*k*, with μ the mutation rate per nucleotide and *k *the length of the sequence in nucleotides. The 2% pairwise sequence divergence defined by DeSalle [57] was used to approximate μ.

## Authors' contributions

CK, LZ and MS analyzed the data, AB, JR and AR planned the research, MS and PS performed the research, CK, LZ and AB wrote the paper and revised the manuscript. All authors read and approved the final manuscript.

## Supplementary Material

Additional file 1 **Sampling sites, geographic coordinates, host pine, collector and haplotype composition of each locality**. The number in brackets after each haplotype name is the number of individuals with that haplotype. Codes refer to the localities shown in Figure 3. Codes for hosts are as follows: PB: *Pinus brutia*; PH: *P. halepensis*; PM: *Pinus mugo/uncinata*; PN: *Pinus nigra*; PP: *P. pinaster*; PR: *Pinus radiata*; PS: *P. sylvestris*; CA: *Cedrus atlantica*, CD: *Cedrus deodara*. Click here for file
