# Supplementary material for: Quaternary history and contemporary patterns in a currently expanding species
Source: BMC Evol Biol. 2009 Sep 4;9:220. doi: 10.1186/1471-2148-9-220 (PMC2753568; doi:10.1186/1471-2148-9-220)
Supplement: Additional file 1 — Sampling sites, geographic coordinates, host pine, collector and haplotype composition of each locality. The number in brackets after each haplotype name is the number of individuals with that haplotype. Codes refer to the localities shown in Figure 3. Codes for hosts are as follows: PB: Pinus brutia; PH: P. halepensis; PM: Pinus mugo/uncinata; PN: Pinus nigra; PP: P. pinaster; PR: Pinus radiata; PS: P. sylvestris; CA: Cedrus atlantica, CD: Cedrus deodara. [file 1471-2148-9-220-S1.pdf]

Locality code	Country	Region / District	Location	# Indiv.	Latitude	Longitude	Altitude (m)	Host	Collector	Haplotype composition	π	H
1	Portugal	Setubal	Alcácer do Sal	12	38º 23’ N	08º 31’ W	40	PP	Teresa Vasconcelos & Manuela Branco	29BA(12)	0	0
2	Portugal	Viseu	Viseu	11	40º 40’ N	07º 54’ W	500	PP	Teresa Vasconcelos	29BA(10) 31BA(1)	0.03%	0.18
3	Portugal	Vila Real	Sevivas	12	41º 31' N	07º 30' W	50	PP	Paola Arnaldo	29BA(5) 30BA(7)	0.088%	0.53
4	Portugal	Braganca	Varges	11	41° 52' N	06° 40' W	400	PP	Paola Arnaldo	29BA(2) 30BA(9)	0.054%	0.33
5	Spain	Southern Iberia	Gibraltar	6	36° 08’ N	05° 21’ W	400	PH	John Cortes	29BB(6)	0	0
6[1]	Spain	Andalusia	Sierra Nevada	12	37° 05’ N	03° 27’ W	1800	PS	José Hodar	1A(10) 3A(2)	0.050%	0.30
7	Spain	Andalusia	Sierra de la Cabra Montés	4	37º 47' N	03º 46' W	1200	PP	Ramon Gonzalez Ruiz	29BA(1) 31BA(3)	0.083%	0.50
8	Spain	Guadarrama	Collado Mediano	5	40° 41' N	04° 02' W	1100	PN PP	INRA	29BA(5)	0	0
9	Spain	Aragón	Boltaña	4	42° 26' N	00° 02' E	650	PS	INRA	1A(4)	0	0
10	Spain	Balearic islands	Sant Llorençt Cardassar	10	39° 37’ N	03° 17’ E	300	PH	INRA	1A(10)	0	0

11	France	Pyrénées Orientales	Osseja	12	42° 23’ N	02° 00’ E	1400	PS PM	INRA	1A(10) 1BG(2)	0.050%	0.30
12	France	Gironde	Pierroton	12	44° 44’ N	00° 46’ W	60	PP	INRA	1A(12)	0	0
13	France	Aveyron	Jouas	3	44° 30’ N	02° 24’ E	430	CD	INRA	1A(3)	0	0
14	France	Haute-Loire	Brioude	12	45° 18’ N	03° 29’ E	600	PS	INRA	1A(12)	0	0
15	France	Loiret	Lorris	12	47° 49’ N	02° 29’ E	150	PS	INRA	1A(12)	0	0
16	France	Alpes de Haute Provence	Thorame	12	44° 04’ N	06° 34’ E	1200	PS	INRA	1A(12)	0	0
17	France	Corsica	Barchetta	10	42° 30’ N	09° 22’ E	100	PP PR	INRA	34BK(2) 34BL(8)	0.059%	0.36
18[1]	Italy	Aosta	Ruines Verrès	26	45° 39’ N	07° 41’ E	1000	PS	University of Padova	1D(23) 1BI(3)	0.035%	0.21
19[1]	Italy	Liguria	Rollo	11	43° 57’ N	08° 08’ E	250	PH	University of Torino	1A(5) 2A(6)	0.18%	0.55
20[1]	Italy	Liguria	Massimino	12	44° 25’ N	08° 26’ E	600	PS	University of Torino	1A(11) 1C(1)	0.028%	0.17
21	Italy	Alto Adige	Silandro	19	46° 38’ N	10° 47’ E	1100	PN PS	University of Padova	1A(17) 1BJ(2)	0.033%	0.20

22[1]	Italy	Friuli	Tugliezzo	12	46° 22' N	13° 11' E	450	PN	University of Padova	1A(6) 1B(6)	0.090%	0.55
23[1]	Italy	Friuli	Venzone	11	46° 20' N	13° 08' E	350	PN	University of Padova	1A(10) 1F(1)	0.030%	0.18
24[1]	Italy	Toscana	M. S. Michele	12	43° 31' N	11° 24' E	890	PN	Univ. of Firenze	1G(12)	0	0
25[1]	Italy	Veneto	Calbarina	12	45° 16' N	11° 43' E	136	PN	University of Padova	1A(12)	0	0
26	Italy	Puglia	Gargano	9	41° 46' N	16° 11' E	200	PH	University of Padova	1G(9)	0	0
27	Italy	Sicily	Nicolosi	12	37° 37' N	15° 01' E	700	PN	University of Catania	1G(12)	0	0
28	Italy	Sardegna	Sanluri	4	39° 33' N	08° 54' E	633	PH	University of Sassari	1G(4)	0	0
29	Italy	Pantelleria	Pantelleria	8	36° 48' N	11° 59' E	190	PP	University of Catania	20AF(8)	0	0
30	Montenegro	Podgorica	Cemovskopolje	4	42° 28' N	19° 17' E	700	PN	M. Glavendekic	33A(4)	0	0
31	Albania	Tirana	Tirana	12	41° 19' N	19° 49' E	90	PN	Fatmir Laceja	1A(12)	0	0

32	Bulgaria	Pirin	Sandanski	8	41° 34’ N	23° 17’ E	220	PN	Daniela Pilarska	1A(7) 1BH(1)	0.041%	0.25
33	Bulgaria	Asenovgrad	Javrovo	10	42° 01’ N	24° 52’ E	700-1000	PN	Plovdiv Station	1A(10)	0	0
34	Greece	Serres	Lailia	2	41º 12´ N	23º 36´ E	900	PN	Maria Kalapanida	1A(2)	0	0
35	Greece	Thessaloniki	Agia Anastasia	3	40º 28´ N	23º 23´ E	50	PB	Maria Kalapanida	1A(3)	0	0
36	Greece	Halkidiki	Skioni	5	39º 58´ N	23º 23´ E	50	PH	Maria Kalapanida	1A(4) 1BE(1)	0.066%	0.40
37	Greece	Thasos	Thasos	11	40° 46’ N	24° 42’ E	na	PH	Maria Kalapanida	1A(8) 1BC(1) 1BD(1) 1BF(1)	0.13%	0.49
38	Greece	Thasos	Potamia	11	40° 43’ N	24° 45’ E	na	PH	Maria Kalapanida	1A(10) 1BD(1)	0.060%	0.18
39	Greece	Samothraki	Samothraki	10	40° 29’ N	25° 31’ E	na	PH	Maria Kalapanida	1A(10)	0	0
40	Greece	Levos	West-Levos	11	39° 10’ N	25° 56’ E	na	PH	Maria Kalapanida	1A(11)	0	0
41	Greece	Levos	East-Levos	10	39° 06’ N	26° 25’ E	na	PH	Maria Kalapanida	1A(9) 32A(1)	0.033%	0.20
42	Greece	Chios	West-Chios	10	38° 22’ N	26° 01’ E	na	PH	Maria Kalapanida	1A(10)	0	0
43	Greece	Chios	East-Chios	11	38° 22’ N	26° 08’ E	na	PH	Maria Kalapanida	1A(11)	0	0
44	Greece	Kardamili	Vassiliki Ft	9	36° 53’ N	22° 19’ E	1400	PN	INRA	1A(9)	0	0
45	Greece	Crete	Chania	12	35° 30’ N	24° 01’ E	na	PH	Maria Kalapanida	17AB(12)	0	0

46	Greece	Crete	Heraklion	9	35° 18’ N	25° 07’ E	na	PH	Maria Kalapanida	15AA(8) 16AA(1)	0.074%	0.22
47	Turkey	Canakkale	Bayramic	2	39° 49’ N	26° 36’ E	300	PB	Mustafa Avci	1A(2)	0	0
48[1]	Turkey	Izmir	Aydin	10	37° 51’ N	27° 50’ E	600	PB	University of Izmir	4E(9) 4H(1)	0.033%	0.20
49[2]	Turkey	Isparta	Gunur	15	37° 46’ N	30° 34’ E	1050	PB PN	University of Isparta	4N(15)	0	0
50[2]	Turkey	Antalya	Karaoz	8	36° 54’ N	30° 43’ E	200	PB	University of Isparta	4N(6) 10P(1) 11N(1)	0.30%	0.46
51[2]	Turkey	Taurus mountains	Pozanti	11	37° 17’ N	34° 51’ E	970	PB PN	University of Padova	12U(7) 12R(4)	0.34%	0.51
52[2]	Turkey	Taurus mountains	Aladag	10	37° 33’ N	35° 22’ E	1100	PB	University of Padova	12K(7) 13K(1) 14W(2)	0.15%	0.51
53[2]	Turkey	Samsun	Samsun	12	41° 17’ N	36° 20’ E	150	PN	Zvi Mendel	4V(12)	0	0
54[2]	Turkey	Iskenderun	Iskenderun	10	36° 34’ N	36° 10’ E	210	PB	University of Padova	12K(5) 12R(3) 12S(1) 12T(1)	0.30%	0.71
55[2]	Turkey	Antakia	Seyhköy	11	36° 04’ N	36° 10’ E	450	PB	University of Padova	5Y (11)	0	0
56[2]	Cyprus	East Cyprus	El Skopi	19	35° 00’ N	32° 40’ E	100-1000	PB PN	Zvi Mendel	6M(1) 7M(9) 7Q(5) 8M(2) 9M(2)	0.15%	0.72

57[2]	Lebanon	Beirut	Beirut	24	33° 53’ N	35° 30’ E	272	PB	American University Beirut	5J(24)	0	0
58	Israel	Golan	Golan Heights	4	32° 58’ N	35° 44’ E	1000	PN	Zvi Mendel	5J(4)	0	0
59[2]	Israel	Upper Galilee	Qiryat Shemona	14	33° 11’ N	35° 33’ E	350	PB	Zvi Mendel	5J(14)	0	0
60[2]	Israel	Lower Galilee	Segev	9	32° 52’ N	35° 14’ E	400	PH	Zvi Mendel	5J(9)	0	0
61[2]	Israel	Judean foothills	Haruvit	15	31° 45’ N	34° 50’ E	150	PH	Zvi Mendel	5J(15)	0	0
62[2]	Israel	S Judean Mts.	Yatir	15	31° 20’ N	35° 03’ E	550	PH	Zvi Mendel	5J(15)	0	0
63[2]	Israel	W Negev	Qisufim	10	31° 22’ N	34° 24’ E	50	PH	Zvi Mendel	5J(10)	0	0
64	Libya	Cyrenaica	Al Bayda	6	32° 45’ N	21° 37’ E	470	PH	University Omar Almukhtar	18AC(3) 18AD(2) 19AE(1)	0.20%	0.73
65	Tunisia	Nabeul	Nabeul	7	36° 27’ N	10° 44’ E	40	PH	M. El Habib Ben Jamâa	21AF(7)	0	0
66	Tunisia	Bizerte	Bizerte	8	37° 02' N	09° 42' E	15	PH	M. El Habib Ben Jamâa	20AF(8)	0	0
67	Tunisia	Dir El Kef	Dir El Kef	7	36° 11’ N	08° 43’ E	370	PH	M. El Habib Ben	20AI(1) 20AG(1) 20AH(4)	0.29%	0.71

									Jamâa	20AF(1)		
68	Algeria	Tellien Atlas	Tikjda	12	36° 00' N	04° 17' E	800-1000	PH	Mohamed Zamoum	22AJ(3) 22AK(5) 22AL(1) 22AM(1) 23AK(2)	0.19%	0.78
69	Algeria	Saharian Atlas	Djelfa Moudjbara	10	34° 30’ N	03° 28’ E	1100	PH	Mohamed Zamoum	26AN(1) 24AN(3) 25AN(6)	0.11%	0.60
70	Morocco	Eastern Middle Atlas	Aknoul	4	34° 40’ N	03° 52’ W	1250	CA	Driss Ghaioule	28AQ(4)	0	0
71	Morocco	Rif	Bab Barred	10	34° 59’ N	04° 50’ W	1300	CA	Driss Ghaioule	28AQ(9) 28AR(1)	0.033%	0.2
72	Morocco	Middle Atlas	Boutrouba	10	33° 27’ N	05° 03’ W	1900	CA	Driss Ghaioule	27AP(10)	0	0
73	Morocco	High Atlas	Oukaimeden	2	31° 17' N	07° 48' W	2300	PP	INRA	25AO(2)	0	0
74	Morocco	High Atlas	Lalla Takerkoust	1	31° 22' N	08° 08' W	750	PH	INRA	25AO(1)	0	0

1. Populations from Salvato et al. *Molecular Ecology* 2002, **11:**2435-2444.

2. Populations from Simonato et al. *Molecular Ecology* 2007, **16:**2273-2283.
